# COVID-19 preparedness and response at a large UK major trauma operating theatres department

**DOI:** 10.1177/1750458920934406

**Published:** 2020-07-08

**Authors:** Carolina R Britton, Gareth Hayman, Claudia Macfarlane, Hemantha Alawattegama, Jasper Ballecer, Nicola Stroud, Alexander Wallace

**Affiliations:** 1 Cambridge University Hospitals NHS Foundation Trust, Addenbrooke's Hospital, Perioperative Care Services, Cambridge, UK

**Keywords:** Theatre utilisation and staffing / Teamwork and communication / Service improvement / Risk management / Leadership / Infection control / Operating theatre

## Abstract

This article aims to describe the early experience of a large major trauma operating theatres department in the East of England during the outbreak of the coronavirus disease 2019 (COVID-19) pandemic. To date and to our knowledge, a small amount of reports describing a surgical department’s response to this unprecedented pandemic have been published, but a well-documented account from within the United Kingdom (UK) has not yet been reported in the literature. We describe our preparation and response, including: operating theatres management during the COVID-19 pandemic, operational aspects and communication, leadership and support. The process review of measures presented covers approximately the two-month period between March and May 2020 and emphasises the fluidity of procedures needed. We discuss how significant challenges were overcome to secure implementation and reliable oversight. The visible presence of clinical leads well sighted on every aspect of the response guaranteed standardisation of procedures, while sustaining a vital feedback loop. Finally, we conclude that an effective response requires rapid analysis of the complex problem that is of providing care for patients intraoperatively during the COVID-19 pandemic, and that retrospective sense-making is essential to maintain adaptability.

**Provenance and Peer review:** Unsolicited contribution; Peer reviewed; Accepted for publication 25 May 2020.

## Introduction and background

Since the World Health Organization (WHO) declared a pandemic on March 11 and at the time of writing this article, there have been 3,925,815 confirmed cases of COVID-19 globally, including 274,488 deaths (WHO 2020) and the UK national status reports indicate 31855 deaths (Department of Health and Social Care & Public Health England 2020). As of May 4, our institution has seen 417 COVID-19 cases with 19% of admissions requiring critical care.

The outbreak of COVID-19 is a significant and urgent threat to global health (Wellcome Trust 2020). Despite that there are ‘no clinical trials to guide practice’ (Gonzalez-Brown et al 2020), a number of articles have been published thus far detailing the experiences of operating theatre departments around the globe (Forrester et al 2020, Gonzalez-Brown et al 2020, Ti et al 2020, Rodrigues-Pinto et al 2020, Wong et al 2020) although none of these are from within the UK.

Our institution follows the increasingly detailed recommendations and tools available from dedicated COVID-19 hubs hosted by both the WHO and by Public Health England (PHE). This guidance informs the management of our operating theatres as a department, and the clinical practice within. At the institution’s wider operational level, command structures were instantaneously formed which jointly devised measures impacting on surgery and the operating theatres. The aims have been to provide surgical care to both pandemic and non-pandemic patients while minimising risks for viral spread and transmission to staff and other patients.

This paper reports the decision-making processes and strategies implemented as a result of the COVID-19 pandemic to date in a department with over 30 operating theatres, employing over 700 staff. The following describes our Standard Operating Procedure (SOP) for preparation and response, including: (1) increasing staffing capability and capacity, (2) shielding and limiting exposure, (3) transmission and infection control measures, (4) operational management and (5) communication, leadership and support. The process review of measures presented covers approximately the two-month period between March and May 2020 and emphasises the fluidity of procedures needed.

## Operating theatres management during the COVID-19 pandemic

### Increasing staff capacity and capability

The WHO (2014) recommends reducing elective surgery as part of planning for surge capacity during a pandemic acute respiratory disease in healthcare. In the current COVID-19 pandemic, such a reduction has already been reported by others keeping the system functioning for both COVID-19 and non-COVID-19 patients (Forrester et al 2020, Wong et al 2020).

Reduction of elective surgery was therefore adopted in our department to decrease the number of in-patients potentially exposed to the spread of the disease within the hospital. It also meant that the burden over the overall services was minimised, and that the ‘social distancing’ measures introduced by the UK government (GOV.UK 2020a) were adhered to. One other aim of reducing elective surgery was to release sufficient medical and perioperative practitioners to: (a) staff 11 to 15 emergency and cancer pathway surgical lists with the additional requirements imposed by ‘infected cases’ (detailed further along in transmission and infection control measures), and (b) to respond to increased demand of staff in order to support critical care.

The ways in which our department increased overall workforce capacity to deal with the pandemic were as follows:

- Redeployment of approximately 80 whole time equivalents (WTE) to critical care wards and critical care surge areas (most notably of nurses and operating department practitioners (ODPs) from the areas of recovery and anaesthesia, as well as theatre support workers (TSWs)).
- Creation of intubation teams covering all areas of the hospital (medical and non-medical practitioners in anaesthesia).
- Deployment of anaesthetic practitioners (circa 12 WTEs) to troubleshoot and maintain anaesthetic machines repurposed for long-term ventilation of patients in critical care.
- Creation of patient ‘proning teams’, assisting with alternating mechanically ventilated patients’ body positioning, a method reported in the literature as effective to improve lung recruitability (Pan et al 2020).
- Establishment of a tracheostomy surgical list.

Besides staff capacity growth, capability increase was also crucial in preparing teams to work in theatres with COVID-19-infected patients as well as in critical care areas. A two-day package of didactic and workshop sessions was developed and delivered by the critical care practice development team and delegates were placed in supernumerary shadow shifts after accelerated induction. Critical care ratio models following the principles of the ‘Guidelines for the Provision of Intensive Care Services’ (The Faculty of Intensive Care Medicine 2019) were maintained to ensure that staff redeployed from theatres had senior supervision from critical care staff.

Facilitation of experiential learning and effective educational feedback were considered vital components of this enterprise. For example, multi-disciplinary simulation sessions for intubation and resuscitation scenarios were repeatedly run by the department of anaesthesia. ‘Patient proning’ twice-weekly workshops were also offered to theatre practitioners. Education and training initiatives further included:

- ‘Donning & doffing’ of personal protective equipment (PPE) drop-in sessions running 8 hours per day for approximately two weeks, attended by surgeons, anaesthetists, theatre practitioners, radiologists and TSWs, as well as others such as endoscopy staff, physiotherapists and clergy personnel.
- Online and remote meeting sessions delivered by company representatives regarding newly acquired anaesthetic machines and their function as long-term ventilators.
- Small group sessions delivered to scrub practitioners to become competent in ‘running’ for anaesthetics (the ‘amber badge’ – so that staff could perform as an ‘amber runner’, described below in ‘transmission and infection control measures’).
- ‘Blood gas analysis’ training for TSWs, enabling them to further assist anaesthetic practitioners in theatres and nursing staff in critical care, while contributing to optimisation of PPE use.
- ‘Ward key nursing skills’ training for registered members of the theatres’ teams temporarily or ad hoc redeployed to wards to help backfill these areas (both online and via one-day group session).
- Daily pre-team-brief 30-minute sessions with multidisciplinary teams focusing on the operating theatre ‘staff and equipment flow’ (described below in transmission and infection control measures).
- The development and contemporaneous dissemination of several guides and education tools (for example: paediatrics COVID-19 intubation checklist; intubation pack checklist; intubation debrief procedure; how to prone and unprone a patient video; COVID-19 ventilation video; etc.) besides those offered by Public Health England (GOV.UK 2020b) and NHS England (NHS 2020).

### Shielding and limiting exposure

Performing individual health risk assessments of all staff working in the operating theatres was treated as a priority. The assessment was guided by trust-wide assessment forms (versions one to four) and supported by Occupational Health Services. The objectives were to shield those healthcare professionals who are extremely vulnerable or at a higher risk (such as those having received a solid organ transplant, the immunodepressed or the severely asthmatic) and allow those at risk (for example pregnancy up to 28 weeks, asthmatic, etc) to avoid contact with COVID-19 suspected or positive patients during their clinical activity. The higher risk group has been working from home where possible or was released on a 12 weeks isolation period, while the medium risk degree practitioners were redeployed to administrative support (namely within education teams) or assigned to work outside ‘red areas’ where full PPE is required.

Limiting exposure is, furthermore, an important part of all planned activities and allocations of staff. It means relieving practitioners as well as creating rosters with the highest rotation possible, so that the same members of staff are not over-exposed by working inside ‘red areas’ uninterruptedly.

### Transmission and infection control measures

Our operating theatres complex employs over 700 staff who work across a total of 37 operating theatres, two of which are located at a satellite unit. Theatres are split into three units, covering trauma and orthopaedics, emergencies, transplant, vascular and general surgery, max fax, plastics, ENT and paediatrics. In addition to this, there are two other complexes with: day surgery, gynaecology and urology, three neurosurgery theatres, two theatres for maternity surgery and two ophthalmology theatres.

Reduction of elective surgery means that only a fraction of the above areas were running. Eleven to 15 surgical lists were therefore allocated to theatres in proximity to each other, as opposed to spread across our several units. Since ‘precautions to prevent human-to-human transmission are appropriate for both suspected and confirmed cases’ (GOV.UK 2020c), the decision to treat all cases coming into the operating theatres as suspected COVID-19 was reached fairly early on in the outbreak. The transmission of COVID-19 is thought to occur mainly through ‘respiratory droplets generated by coughing and sneezing, and through contact with contaminated surfaces’ (GOV.UK 2020d). Furthermore, during aerosol-generating procedures (AGPs), there is an increased risk ‘spread of infectious agents (…) and airborne precautions must be implemented’ (GOV.UK 2020d). Specification of procedures considered to be AGPs can be found in national PPE guidance (GOV.UK 2020e). In addition, ‘all secretions (except sweat) and excretions, including diarrhoeal stools from patients with known or possible COVID-19, should be regarded as potentially infectious’ (GOV.UK 2020f). In the operating theatres, consideration must also be given to the formation of fomites, objects that become contaminated with infected organisms and which subsequently transmits those organisms to other people (such as surfaces, medical equipment, keyboards or any inanimate object) (NHS England 2020a).

As recommended by the guidance cited so far (GOV.UK 2020e), our SOP stipulates that:

- Both conventional ventilation and laminar flow remain fully on during surgical procedures to ensure rapid dilution of aerosols and to protect operating room staff, and since ‘air passing from operating theatres to adjacent areas will be highly diluted and is not considered to be a risk’.
- Theatres are informed in advance of a patient transfer of a confirmed or possible COVID-19-positive case and the patient is transported directly wearing a surgical mask when tolerated.
- Patients are anaesthetised in theatre (not in the anaesthetic room, as standard) and staff wear protective clothing (including Filtering Facepiece Particles (FFP) 2 or 3 respirators) as per national guidance.
- Instruments and devices are decontaminated in the normal manner in accordance with manufacturers’ advice.
- Theatre rooms are cleaned as per local policy for infected cases (Tables 1 and 2).
- AGPs are carried out in the operating single room with the doors shut and only those healthcare staff who are needed to undertake the procedure are present.

**Figure 1:**
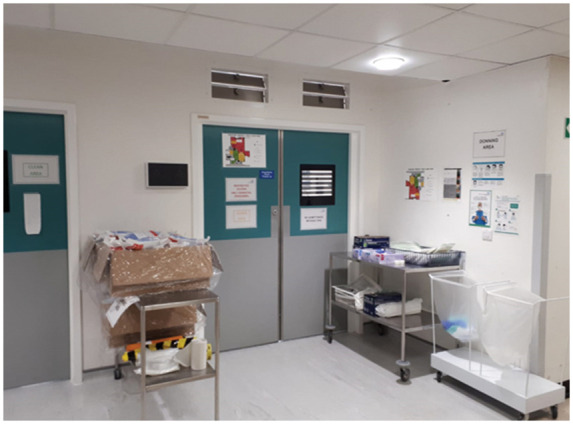
Example of designated area for putting on PPE

**Figure 2:**
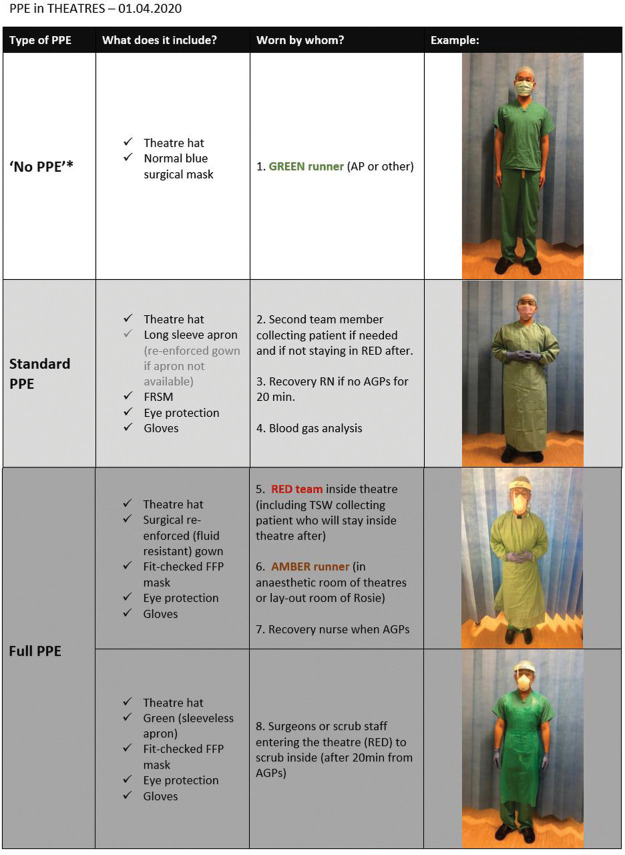
PPE in the operating theatre

**Figure 3:**
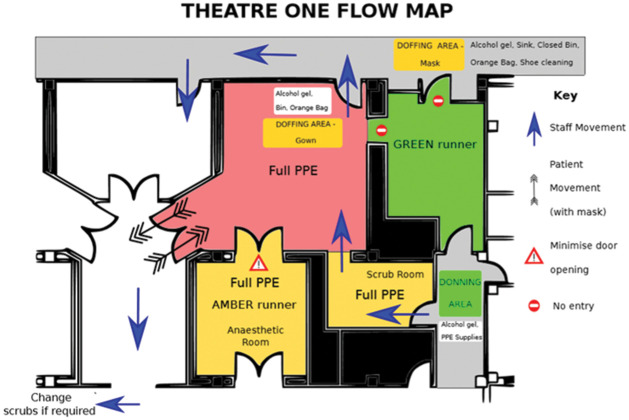
Example of theatres’ flow maps

In addition to the above, measures concerning PPE protocols and operating theatres’ flow were also put in place. Designated areas for donning were identified outside of the theatre rooms, separated by a door and with enough space to store PPE and for a minimum of two people to don simultaneously (Figure 1). Doffing of gown and gloves occurs inside the theatre rooms and the masks are removed once in designated area outside (Figure 1). Guidance and infographics were displayed appropriately (Figure 2). Theatres’ flows and different zones were also defined using the areas’ blueprints, for infection control purposes but also to ensure that activities in theatres were safe and as productive as possible. Figure 3 depicts an example of a theatre flow map. These images were displayed at the entrance of each area (as seen in Figure 1) and a laminated copy is still currently used during the routine walk-throughs led by the practitioner in charge of the operating theatre, prior to team brief, ensuring that all members of the team are aware of the area flow.

The areas include:

*Zone 1 (green)*: the corridor-type area where donning stations were established and where staff put on PPE.

*Zone 2 (amber, no admittance without PPE)*: the scrub room, typically an ante-room to the operating theatre itself, where surgical staff ‘scrubs’ and put on sterile gown and gloves.

*Zone 3 (amber, no admittance without PPE)*: the anaesthetic room, where the registered practitioner wearing full PPE performs the ‘amber runner’ role, in order to supply extra required equipment, drugs, blood gas results, etc.

*Zone 4 (red: no admittance without PPE, only essential personnel)*: the operating room itself.

*Zone 5 (amber to green, no re-entry)*: the exit route, where PPE is removed and from which there is no re-admittance into the operating room.

## Operational management

Detailed workflows, defining the roles of each member of the team during the various phases of care of the patient in theatres, have been published by others (Gonzalez-Brown et al 2020, Ti et at 2020) and the following describe our departments’ specific experience.

### Theatre personnel requirements

For all patients coming to theatres, anaesthesia is induced inside the theatre itself (bypassing anaesthetic room), by a team of one or two anaesthetists (depending on anticipated complexity of case), besides one AP and one TSW. During the operating procedure, they are joined by one or two scrub staff (depending on nature and length of procedure) and the surgeons. In the anaesthetic room (zone 3), a registered member of staff (nurse or operating department practitioner) circulates within to provide controlled or other drugs and bridge supply of extra surgical or other equipment required from inside the operating room. This ‘amber runner’ is provided with a walkie-talkie to communicate with the external (‘green’) runner to minimise the number of times the anaesthetic room door is opened during the procedure. The latter runner remains in the ‘green’ areas (zone 1), where PPE is not required except to run blood gas analyses. Recovery of the patient in theatre is assisted by one recovery staff member and one TWS or scrub practitioner, wearing full PPE due to the potential for suctioning or any other AGPs post-extubation.

### WHO surgical safety checklist and special considerations during team brief

The WHO surgical safety checklist (WHO 2009) was adapted locally to include:

- Determining if a second anaesthetist is required for the case.
- A pre-team-brief walk-through so that each member of the team is familiarised with the red, amber and green zones, as well as the donning and doffing areas and procedures.
- Determining each member’s role in relation to the defined green-amber-red areas.
- Reminding all of the team of the need to wait for indication to enter after that the airway is secured (20 minutes after AGP).
- Identification of all back-up surgical and anaesthetic equipment that may be required in order to minimise traffic through the amber area (zone 3).
- Confirm postoperative care arrangements.
- Decide on rest periods and plan accordingly.

### Considerations for preparation of theatre

Preparation of sterile instruments and equipment is done prior to sending for the patient and empty containers are kept outside the theatre room until they receive the dirty sets from the scrub practitioner at the end of the procedure. The anaesthetic machine is checked by the anaesthetist and AP, as well as all the airway equipment and drugs are drawn up and moved to inside the theatre. The AP sets up the circuit with the mask and High-Efficiency Particulate Air (HEPA) filter attached to the catheter mount and a Heat and Moisture Exchanger Filter (HEMF) on anaesthetic machine expiratory outlet.

Scrub staff set up inside the theatre before the patient is sent for, and leave prior to the patient’s arrival. This is done to optimise the use of PPE (as FFP masks can be only be used for limited length of time, as per manufacturer’s recommendations). After final check with the anaesthetic team, the patient is collected from the ward and both the anaesthetist(s) and AP don full PPE using each other as ‘donning buddies’, to assist with checking correct technique as per recommended (GOV.UK 2020f).

### Patient preparation in ward and transport to theatres

Patients transferred from the ward into theatres are asked to wear a fluid-resistant surgical mask (FRSM) if they can tolerate, and when necessary fitting over oxygen delivery device which may include nasal prongs, a Hudson mask or a non-rebreather mask up to 15 litres for transfer. Any wounds the patient may have are covered to ensure body fluids are contained. Patients wash or disinfect their hands before leaving their rooms when possible and wear a clean gown or robe and are covered by a clean sheet or drape for transport if required. Finally, any paperwork accompanying the patient is transported in a plastic wallet to prevent contact with the patient or contaminated linen.

Collection of the patient is performed by the TSW (‘red runner’) or the registered scrub practitioner, who takes their full PPE with them (including FFP 2 or 3 mask) in order to don at the designated area in ward and stay with the patient inside the operating room during the intubation procedure. One other member of the team assists with the collection and transfer of the patient, who should be the ‘green runner’, wearing protective gown, gloves, visor and FRSM only (henceforth referred to as ‘standard PPE’). In order to allow these practitioners to refrain from touching the environment (elevator buttons or swipe card access), a member of the transport team with ‘clean hands’ is required.

### Considerations for intraoperative period

The ‘red runner’ who collected the patient from the ward takes him or her straight to the operating theatre and the sign-in stage as per the WHO surgical safety checklist ensues. The anaesthetist and AP, besides the ‘red runner’ will be wearing full PPE and the surgeon(s) wear standard PPE, as they will leave the theatre via the doffing area after assisting with transfer on to the surgical table. The patient’s bed is either kept inside the theatre until the patient leaves to the postoperative ward where there is sufficient space for this, or is removed and stripped of linen (placed in red alginate bag) and immediately cleaned according to the local cleaning protocol (Table 2).

**Table 1:** Theatres’ cleaning procedure

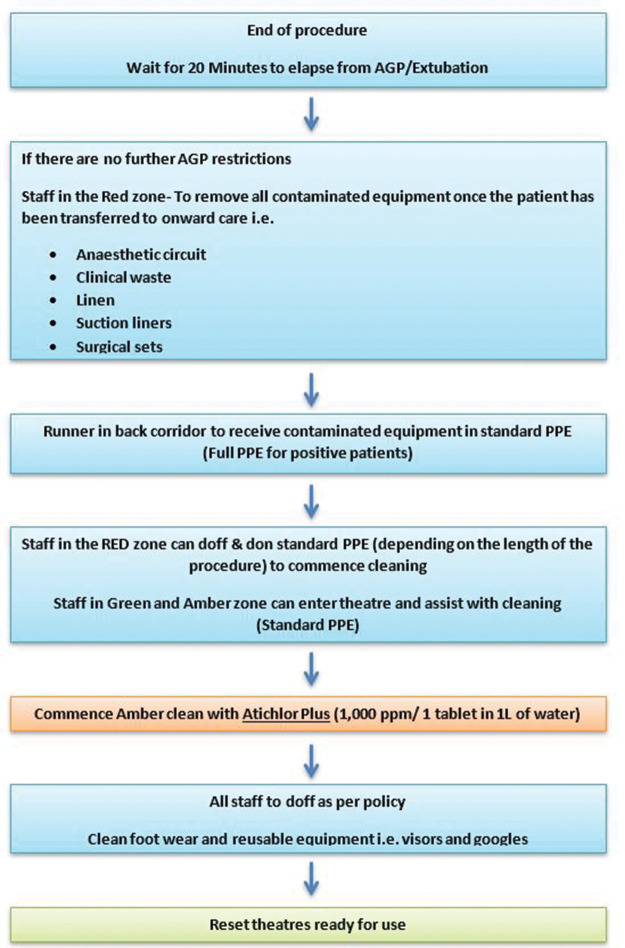

**Table 2:** Theatres’ infection control infographic

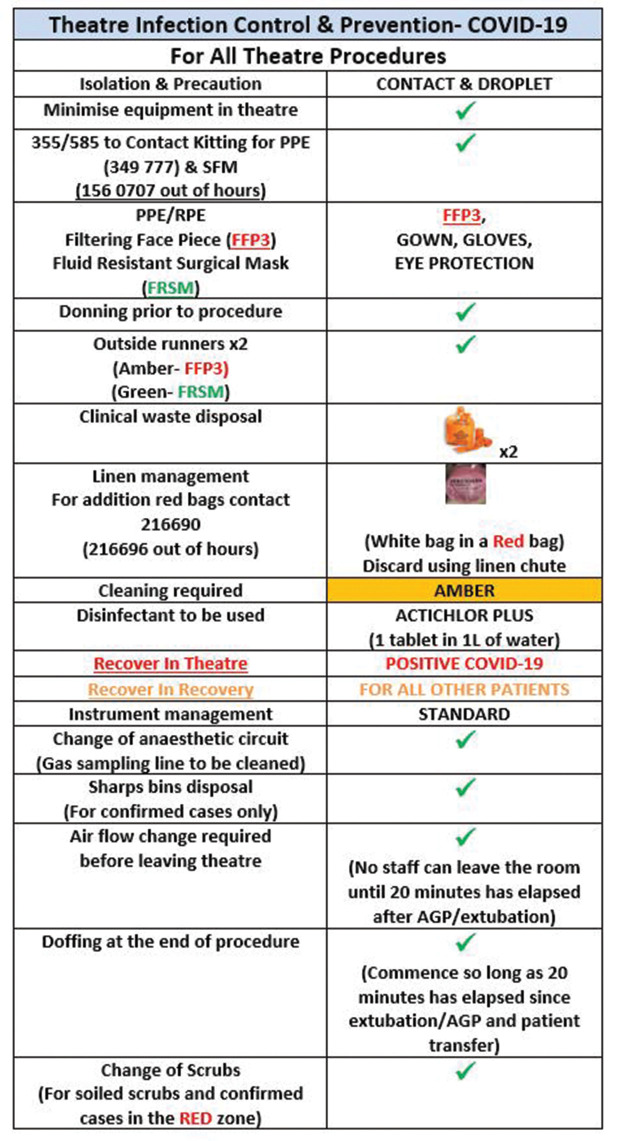

Upon induction of anaesthesia, the operating theatre becomes a ‘red zone’ and no staff are allowed to enter or exit the area for 20 minutes post-intubation. Twenty minutes is the length of time required for the clearance of aerosols given the number of air changes per hour in our theatres, following national guidance (Public Heath England & National Health Service 2020). The time for permitted entrance is noted and written down on a laminated ‘stop sign’ which is placed on the inside of the window of the theatre room. If required, surgical staff can enter the room in full PPE via the designated entrance (ie: zone 2 described above).

### Considerations for end of procedure (include cleaning and recovery)

The time of the last AGP is noted and surgeon(s) exit the theatre if there are no AGP restrictions and ensuring that an adequate number of people remain (following our standard manual handling protocol) for transfer of the patient onto the bed for extubation. The documentation of surgical notes takes place in computers outside of the theatre. Surgical drapes are removed carefully to avoid spread of contaminated fluids. Surgical instruments remain inside the theatre room. After the patient is transferred, any members of staff besides those required for extubation leave the room via the doffing route. No entry or exit is allowed until 20 minutes have elapsed after extubation.

All known COVID-19-positive patients are recovered in theatres with the presence of a recovery practitioner. All other patients are recovered in the recovery unit. After the handover to the recovery practitioner, the anaesthetic team exits the theatre so long as 20 minutes have passed since the last AGP. Full PPE including FFP 2 or 3 masks are required for staff recovering the patient because continuous positive airway pressure (CPAP) and high-flow nasal oxygen (HFNO) are considered AGPs, as well as upper airway suctioning manoeuvres.

All staff doff in designated area and as per national guidance (GOV.UK 2020f). A shower and/or change of scrubs are not recommended unless the practitioner feels it is necessary due to the nature of procedure and own exposure.

### Considerations regarding emergency in theatre

Emergency situations in theatres may occur in relation to the patient or if a member of staff feels unwell. In the first case, the emergency button is pressed, and the ‘amber runner’ is to transmit to the outside what is the nature of the emergency and/or who is required to assist. No one is to enter without having donned appropriate PPE through the designated entrance. If help is required to assist a member of staff, the same principles apply, and the utmost care should be taken in removing PPE from collapsed staff members. Cleaning protocols need to be extended to areas momentarily compromised by safe removal of staff members requiring assistance.

## Communication, leadership and support

The development and implementation of the COVID-19 epidemic operating theatres SOP, of which the above measures are but a part, required: extensive review of all published guidance and multi-disciplinary discussion to reach consensus on all decisions. Furthermore, input from all staff groups on the requirements for clarification and standardisation was also necessary. Maintaining open and effective routes of communication in such a large department required a significant effort from all. The most significant methods through which communication was achieved include:

- The surgical units 2-minute morning briefs (extended to 10 minutes).
- The two daily 30-minute meetings between all team leads, operations manager, theatres’ matrons, emergency coordinator, administration consultant of the day and sterile services coordinator.
- Daily updates from the trust corporate office.
- Email inboxes monitored and actioned upon by both the practice education team and the anaesthetics department to ensure timely reply to practitioners.
- Publication in shared folders, notice boards and in-theatre documentation folders of all guidance and SOP measures adopted in real time.

In addition to these forms of communicating and cascading down information as well as escalating concerns, the above-mentioned command and control centres hold daily meetings or conference calls ensuring timely response to the evolving situation of the COVID-19 pandemic both from the department and from the wider institution.

Peer support and leadership action have been crucial for the development and implementation of our SOP, as discussed in more detail further down. Most importantly, opportune emotional and psychological support was made available to practitioners, including: 24-hours confidential helpline, qualified coaches’ confidential meetings, psychological support service, professional wellbeing and clinician support, as well as chaplaincy and the ‘freedom to speak up’ service. Other programmes such as TRE (trauma/tension release exercise) sessions as well as the internal ‘critical care psychological wellbeing service’ are also offered to staff, in particularly those redeployed to COVID-19 ‘red areas’, from within our department. National NHS staff support initiatives, as well advice as from the Royal Colleges and other Associations were also extensively shared.

## Discussion

Even while writing this article, modifications to national policy regarding PPE usage have been published (GOV.UK 2020g). The importance of optimising PPE use while keeping everyone safe will have implication in the staffing of operating theatres, and possibly on the classification of urgency for surgical treatment prior to testing for COVID-19. As such, our department’s ‘COVID-19 pandemic SOP’ is already under review for version 2 release and will include changes to the above described due to constantly changing requirements, while maintaining standards of care and safety. The paragraphs that follow attempt to give an idea of what it has meant, so far, to deal with complex problems in our department.

Firstly, reassuring staff of the rationale behind every action and protocol adopted was deemed as important as any other measures put in place to keep everyone safe and able to carry on with their clinical work. In this context, leadership within the operating theatres was crucial. In our department, team leads are expected to be effective managers of resources and of the care environment and to lead flourishing teams in a learning culture, as defined by the organisation’s key performance indicators (KPIs) for theatres team leads. The COVID-19 epidemic constitutes a great threat to team cohesion and concerted work. Major workforce re-shuffling and non-trivial sickness/isolation also meant great pressure, as teams became depleted of many staff members.

Secondly, we have found that fuelling high-performing teams in the current pandemic requires leadership resilience. There is a plethora of theories and conceptual frameworks from the social, cognitive and behavioural sciences that can assist in interpreting phenomena in ‘group dynamics’, a term coined by Kurt Lewin with roots in Gestalt psychology (Hogg & Williams 2000). Our experience in belonging to a team – ‘sharing a common goal, as well as interdependent actions and outcomes while embedded in our own organisational context’ (Devine 2002) – during the COVID-19 crisis, undoubtedly deserves close attention and reflection. Unsurprisingly, ‘forming–storming–norming–performing’ phases of group development (Tuckman 1965) were rebooted and aborted during the last few weeks, as teams themselves were pushed to change makeup (for example due to unexpected sickness) or methods of working together at very fast tempo.

Furthermore, proper coordination of emergency response efforts has been ensured by command and control activities, as is necessary in all crisis management situations (Geneviève et al 2010) but the efforts to channel teams’ energy to the tasks at hand also require astute leadership from our operating theatres practitioners. The most useful insight is that, in order to become a functional again, teams need to overcome ‘emotionality, (…) followed by a sense of “pulling together” in the group and of being more sensitive to one another’ (Tuckman 1965: p396).

Therefore, the feasibility of our responsive actions was evaluated together with how teams functioned. Implementation and reliable oversight, furthermore, depended on team leads being present, approachable and methodically informed. Our teams’ initiative in self-assessing clinical human factors impact on performance has most likely positively influenced this adaptability and cohesion, meriting further investigation. Further details of this initiative can be found in ‘The Face of AfPP Competition, April winner’ article (AfPP 2020).

Our department’s response to the pandemic demanded handling of several complicated and complex issues, as others have also acknowledged (Forrester et al 2020, Rodrigues-Pinto et al 2020). Understanding the complexity of the domain of healthcare in different contexts is an under-explored area (Mark 2006). However, in order to disallow disorder, team leads formed a close-knit group to make decisions in a way resembling the Cynefin framework (Cognitive Edge 2010) for handling problems from simple to complicated, complex or chaotic. It has been argued that the Cynefin framework provides a ‘sense-making framework that is inclusive and non-hierarchical’ (Mark 2006: p.863), arguably an essential tool for compassionate leadership (West 2019).

## Conclusion

In this article, we aimed to document our account of something scarcely reported in the literature, hoping that it can constitute a useful contribution to other settings and systems responding to the COVID-19 pandemic, particularly in the context of operating theatres.

We conclude that a rapid and flexible COVID-19 pandemic response of a large complex of operating theatres depends on cohesive teamwork and communication and rapid sharing of both information and rationale for strategies adopted. We emphasised theatre practitioners’ leadership and strategies for sense-making as crucial to maintain effectiveness.

Finally, as other settings’ experiences come to light, high quality empirical research is needed to find ‘right answers’, so that preparing and responding to a pandemic of a respiratory virus such as the SARS-CoV-2 can become part of healthcare’s knowable, albeit only partially understood, domain of problems.
